# Feasibility of a randomized clinical trial evaluating a community intervention for household tuberculosis child contact management in Cameroon and Uganda

**DOI:** 10.1186/s40814-022-00996-3

**Published:** 2022-02-11

**Authors:** Anca Vasiliu, Georges Tiendrebeogo, Muhamed Mbunka Awolu, Cecilia Akatukwasa, Boris Youngui Tchakounte, Bob Ssekyanzi, Boris Kevin Tchounga, Daniel Atwine, Martina Casenghi, Maryline Bonnet, on behalf of the CONTACT study group, Maryline Bonnet, Anca Vasiliu, Savine Chauvet, Elisabete de Carvalho, Sayouba Ouedraogo, Georges Tiendrebeogo, Martina Casenghi, Jennifer Cohn, Boris K. Tchounga, Boris Y. Tchakounté, Collette Sih, Rogacien Kana, Eric Youm, Patrice Tchengou, Léonie Simo, Paul W. Manguele, Paul Bindzi, Marie-Louise A. Ndongo, Doline Ndjang Kombou, Jinette L. Guedem Nekame, Narcisse Sitamze Kaptue, Philippe N. Tsigaing, Muhamed M. Awolu, Leticia G. Seuleu Ndjamakou, Naomi Chi Ndum, Daniel Atwine, Bob Ssekyanzi, Rinah Arinaitwe, David Otai, Cecilia Akatukwasa, Joanita B. Tebulwa, Hamidah Kamanzi, Agnes Natukunda, Eva Natukunda, Rose Kyarimpa, Doreen Kyomuhendo, Scovia Sanyu, John Ssemanya, Richard Okello, Albert Kuate Kuate, Stavia Turyahabwe, Stephen M. Graham, Peter J. Dodd, Nyashadzaishe Mafirakureva, Sushant Mukherjee

**Affiliations:** 1 University of Montpellier, IRD, INSERM, TransVIHMI, Montpellier, France; 2 Elizabeth Glaser Pediatric AIDS Foundation, Yaoundé, Cameroon; 3 Epicentre Research Center, Mbarara, Uganda; 4 Elizabeth Glaser Pediatric AIDS Foundation, Geneva, Switzerland

**Keywords:** Pediatric tuberculosis, Community intervention, Tuberculosis preventive therapy, Tuberculosis screening, Active contact investigation, Feasibility, Acceptability, Mixed methods, Cluster randomized trial, Complex intervention

## Abstract

**Background:**

One of the main barriers of the management of household tuberculosis child contacts is the necessity for parents to bring healthy children to the facility. We assessed the feasibility of a community intervention for tuberculosis (TB) household child contact management and the conditions for its evaluation in a cluster randomized controlled trial in Cameroon and Uganda.

**Methods:**

We assessed three dimensions of feasibility using a mixed method approach: (1) recruitment capability using retrospective aggregated data from facility registers; (2) acceptability of the intervention using focus group discussions with TB patients and in-depth interviews with healthcare providers and community leaders; and (3) adaptation, integration, and resources of the intervention in existing TB services using a survey and discussions with stakeholders.

**Results:**

Reaching the sample size is feasible in all clusters in 15 months with the condition of regrouping 2 facilities in the same cluster in Uganda due to decentralization of TB services. Community health worker (CHW) selection and training and simplified tools for contact screening, tolerability, and adherence of preventive therapy were key elements for the implementation of the community intervention. Healthcare providers and patients found the intervention of child contact investigations and TB preventive treatment management in the household acceptable in both countries due to its benefits (competing priorities, transport cost) as compared to facility-based management. TB stigma was present, but not a barrier for the community intervention. Visit schedule and team conduct were identified as key facilitators for the intervention.

**Conclusions:**

This study shows that evaluating a community intervention for TB child contact management in a cluster randomized trial is feasible in Cameroon and Uganda.

**Trial registration:**

Clini calTr ials. gov NCT03832023. Registered on February 6^th^ 2019.

**Supplementary Information:**

The online version contains supplementary material available at 10.1186/s40814-022-00996-3.

## Key messages regarding feasibility

- What uncertainties existed regarding the feasibility?

We were uncertain about the possibility to recruit the necessary sample size from the study clusters in 12 months. We did not know if a community intervention for tuberculosis screening and preventive therapy management would be acceptable by providers, beneficiaries, and their communities. We wanted to better integrate this complex intervention into existing tuberculosis services in order to facilitate its programmatic scale-up at the end of the research study.

- What are the key feasibility findings?

We found that we would need to extend the recruitment period to 15 months in order to reach the sample size. Discussions with patients, health staff, and community showed that the community intervention is acceptable as long as confidentiality is respected, counseling is provided, and the staff delivering the intervention are well trained. Findings from the tuberculosis services survey allowed us to better adapt and integrate study activities into existing services.

- What are the implications of the feasibility findings for the design of the main study?

We used the findings of the feasibility study to fine-tune the community intervention in order to implement and evaluate it in a manner which is respectful to the local context and can easily be scaled-up.

## Background

Tuberculosis (TB) is a preventable and curable disease. Nonetheless, the World Health Organization (WHO) estimates that more than one million children develop TB disease every year, representing 12% of the global TB burden [1]. Africa carries a high burden of TB disease, with 25% of global new cases occurring in this region. The majority (80%) of children dying from TB are younger than 5 years old [2], and mathematical models show that 96% of them die before treatment, mainly because they were not diagnosed with TB [2]. One of the main transmission pathways for children takes place in the household, usually from a caregiver or another adult present in the household [3, 4]. When infected, children progress more rapidly towards TB disease and often present with severe forms of TB, especially if they are young (less than 5 years) or HIV-positive [5, 6].

To increase early detection, WHO recommends for all children living in the same household with a bacteriologically confirmed adult TB patient, to be screened using at least a symptom-based screening. Those with a negative TB screening, with a priority given to young or HIV-positive children, could then be initiated on tuberculosis preventive therapy (TPT) to prevent progression to TB disease [7, 8]. Nevertheless, WHO estimates that only 33% of estimated eligible contact children were started on TPT in 2019 [1]. Health system and patient-related challenges [9–12] have already been described regarding contact screening and TPT initiation in resource-limited settings. Among them, one major challenge is the necessity for caregivers to bring children who may not have any symptoms to the health facility for TB screening and to bring them back on regular appointments for follow-up if they were initiated on TPT, knowing they are healthy children.

Previous findings from the literature show that community interventions have improved TB treatment outcomes [13, 14] and that involving community healthcare workers (CHW) had a great impact on TB case finding [15–17]. Community interventions can also increase the coverage of TB screening and initiation on TPT among household child contacts. There has not been any randomized controlled trial (RCT) comparing the effectiveness of a community intervention for TB screening and TPT management to a facility-based intervention. Our research group is conducting a pragmatic cluster RCT (cRCT) evaluating a community intervention for household child-contact management in Cameroon and Uganda. Both countries have high TB incidence of 179 and 200/100,000 population for Cameroon and Uganda, respectively. The TPT coverage is relatively low in both countries, 43% in Cameroon and 34% in Uganda [1]. The CONTACT study (Community iNtervention for TB Active Contact Tracing and preventive therapy management) is part of the CaP TB Project (Catalyzing Pediatric Tuberculosis Innovations), a multi-country project aimed at improving pediatric TB case finding and access to TPT through a multipronged approach including implementation of decentralized and integrated models of care, capacity building of front line health care workers on management of pediatric TB, improved access to timely and accurate diagnosis, and effective treatment for active TB disease and TB prevention [18]. The CONTACT study is composed of three phases, (1) pre-intervention phase: feasibility study and aggregated data collection; (2) intervention phase: participant inclusion; and (3) evaluation phase: effectiveness, cost-effectiveness, process evaluation.

Under the CONTACT study, household child-contacts of bacteriologically confirmed index cases are being screened for TB at the household, and children under 5 years old or HIV-positive are also initiated on TPT if asymptomatic and followed-up in the household by CHW. Only symptomatic children or children facing safety issues with TPT are referred to the facility. More information can be found in the previously published study protocol [19].

Complex interventions are like black boxes more often than not and important processes and decision-making in the early stages of intervention development are seldom reported [20]. Before evaluating a complex [21] community intervention, it is crucial to assess the feasibility of the proposed intervention to orient and prepare the investigators for full-scale research [22, 23]. This is particularly essential in case of (1) activities needing any sort of community involvement and partnership, (2) when the data available in the literature are scarce regarding a specific technique or intervention, (3) when the population has specific socio-cultural differences and specificities, and (4) when available literature is described in different settings (e.g., high-income countries) [23]. The first three criteria apply to the proposed community intervention of the CONTACT study.

Therefore, the main objective of this study was to assess the feasibility of a community intervention for TB household child contact management and the conditions for its evaluation in a cRCT in two high-burden, resource-limited countries, Cameroon and Uganda.

The specific objectives of this study were to assess (1) recruitment capability of study sites, (2) acceptability of the intervention by beneficiaries and providers, and (3) adaptation, integration, and resources of the community intervention in the health system organization, using a feasibility framework proposed by Orsmond and Cohn [24].

## Methods

### Study design

This feasibility study used a convergent design based on concurrent quantitative and qualitative data collection and analysis, including with focus group discussions (FGD) and in-depth interviews (IDI), retrospective cohort, survey, document review, and expert discussions (Table 1).

**Table 1:** Outcomes and data collection methods of each feasibility dimension

	Recruitment capability	Acceptability	Adaptation, integration and resources
Outcome	Number of bacteriologically confirmed tuberculosis cases per cluster Number of children < 5 per household	Perceptions and opinions of the people receiving and delivering the intervention	Routine pediatric tuberculosis activities Availability of existing resources (human resources, registers, drugs, diagnostics)
Data collection	Retrospective cohort from TB registers from April 2018 to March 2019 Review of Demographic Health Survey	Focus group discussion with beneficiaries In-depth interviews with healthcare providers and community members	Cross-sectional survey of tuberculosis services at cluster sites Discussions with National Tuberculosis Program and CaP TB representatives Review of national policy and guidelines
Data collection period	September-October 2019	July-August 2019	July-September 2019

*CaP TB* Catalyzing pediatric tuberculosis innovations

Data were collected concurrently during the preparation phase of the CONTACT study, 3 months before the start of inclusions from July to October 2019 as presented in the feasibility timeline in Additional file 1.

### Study setting

The healthcare system and TB service provision are different in Cameroon and Uganda. TB services in Cameroon are delivered mainly in centers for diagnosis and treatment in district or regional hospitals, whereas in Uganda, TB services are decentralized down to primary health centers (PHC). Community activities are common in Uganda for TB patients’ treatment follow-up, whereas in Cameroon, TB activities are mainly facility-based and community interventions are mainly on HIV, family planning, and malaria. In Cameroon and Uganda in 2019, there were an estimated 27% and 29% of household contact children < 5 years on TPT, respectively (1).

The CONTACT study is implemented in 20 clusters, 10 in each country. A cluster is defined as a health facility being part of the CaP TB Project and its catchment area to ensure the availability of similar diagnostic tools for presumptive TB children across clusters sites. In Cameroon, the clusters are district hospitals identified in 10 districts from two regions (Centre and Littoral) and in Uganda, clusters are 13 PHCs and 2 hospitals (more than one facility per cluster) in 4 districts from one region (South-West). Rural or semi-urban clusters are the main focus of the intervention [19]. The primary outcome of the CONTACT study is the proportion of child contacts < 5 years of adult bacteriologically confirmed TB cases who initiate and complete TPT with a sample size of 1500 contact children < 5 years, which represents a minimum of 75 participants per cluster. Taking into account a cluster size variability of 50%, a minimum of 50 participants per cluster was required with an objective to complete enrolment in 12 months. The clusters were selected from the facilities supported by the CaP TB project in two regions of Cameroon and one region in Uganda in order to ensure a similar level of pediatric TB case management across study sites. However, this limited the number of eligible sites.

### Recruitment capability

In the absence of information about the expected number of contact children < 5 years in the study sites, we chose a proxy for estimating the study population size. The most practical proxy was to estimate the number of children < 5 years per bacteriologically confirmed adult index case . We searched the relevant data from the Demographic Health Surveys [25, 26] of each country and the relevant literature on TB patients’ household sizes [27].

To estimate the number of index cases per cluster, study research assistants (RAs) retrospectively collected aggregated data from the National Tuberculosis Program registers from March 2018 to April 2019 in all study cluster facilities using a REDCap data collection tool [28]. Information about the type of TB (pulmonary or extrapulmonary), bacteriological confirmation, age of TB patients, their HIV status, and their treatment outcomes were collected following a standardized operating procedure, and data was monitored at the end of the activity. We compared these data with aggregated data provided by the National TB Program from 2017 from the same cluster facilities to assess potential variability in TB detection throughout the years.

### Acceptability

We conducted a qualitative assessment in 4 clusters of two regions (Centre and Littoral) in Cameroon and 2 clusters of one region (South-West) in Uganda. In both countries we used the same discussion guides which was tested before the qualitative activities with a community leader and a facility manager for the comprehensiveness of the guide and on the qualitative informed consent from clusters that were not selected for the qualitative study. Focus group discussions (FGDs) with a minimum of 6 TB patients with household child contacts were conducted separately among women and men. In each selected site, in-depth interviews (IDIs) were conducted with the facility TB focal person and manager, a community leader identified by the TB focal person, and one of the community healthcare workers collaborating with the facility. FGD participants were randomly selected from the TB registers by the facility TB focal person by selecting retrospectively every 5^th^ registered patient until nine (maximum number) participants accepting to come to the health facility for the FGD.

FGDs and interviews were conducted in French in Cameroon, and in English or Runyankole in Uganda. The qualitative research team recorded all discussions and verbatim transcriptions were done for all recordings and validated by a different researcher for consistency against audio files.

### Adaptation and integration

#### TB services and existing tools

Research assistants or CaP TB programmatic officers filled a standardized questionnaire using data from the facility baseline assessment done by the CaP TB program and that were completed by discussions with the facilities’ TB focal persons about (1) child contact investigation, TB screening, and diagnosis in cases of presumptive TB and TPT services (drugs, dosages, and mode of delivery); (2) TB/HIV management (integration of services); (3) the referral system between different levels of health care facilities; (4) drug management; and (5) TB case recording tools used under routine and implemented by the CaP TB project (see Additional file 2 for the data collection tool).

#### Data quality check

To assess if the National TB Program facility TB registers represent a reliable source of data collection for index cases, we quantified missing data and errors between May 1^st^ 2018 and Oct 31^st^ 2018 on key variables. Research assistants verified the TB register for the following fields: registration date, TB registration number, sex, age, type of TB, type of patient (new/retreatment), and HIV status, using a standardized data collection tool according to a standard operating procedure describing procedures for verifying TB register data accuracy by cross-checking the TB register with other facility documents (TB laboratory register, patients’ files, and TB treatment cards). Missing data was any field which was not filled in and error was erroneous data after verification of the source of the information. All data were monitored.

#### Resources and procedures

This part of the feasibility assessment was focused on identifying eventual logistic, programmatic, or financial gaps with possible solutions, and then ensuring that all human resources, operational, and logistical prerequisites were met for an optimal implementation of the intervention. For procedure development, the team reviewed the National Guidelines on TPT management [29, 30], National TB or pediatric TB guidelines [29, 31], WHO Integrated Management of Childhood Illness guidelines [32], WHO latent TB guidelines [7, 33], and WHO pediatric TB guidelines [8].

#### Data analysis

The number of bacteriologically confirmed patients of 15 years and above was reported per month and facility over a 1 year period. The expected number of children under 5 years per household was estimated from the percentage of children under 5 years per household and the mean household size, both reported in the demographic health survey of each country. The missing and error rate of predefined variables of bacteriologically confirmed patients from the TB register was calculated as the number of missing and errors divided by the total number of individuals registered. Medians of the missing and error rate were presented with the interquartile range (IQR). The qualitative data was coded using an axial coding strategy, regrouping the codes in categories and categories in themes using Atlas.TI version 9.0.

#### Ethics

This feasibility study was part of the main cRCT protocol that has been approved by the WHO Ethics Research Committee, the Advarra Institutional Review Board and by the two local ethics committees: Cameroon National Ethics Committee for Human Health Research and Research Ethics Committee of the Mbarara University of Science and Technology in Uganda. In addition, administrative approvals were needed from the Direction for Operational Research from the Ministry of Health in Cameroon and the Ugandan National Council for Science and Technology in Uganda. Participation in the qualitative assessment of acceptability was voluntary and all participants signed an informed consent form before discussions or interviews.

## Results

### Recruitment capability

The review of the Demographic health survey DHS data [25, 26] in Cameroon identified 16.9% children < 5 years per household and a mean of 5 household members, which represented 0.85 children < 5 years per household. In Uganda, there were 18% children < 5 years per household, with a mean of 4.5 household members, which corresponded to 0.81 children < 5 years per household.

**Fig. 1:**
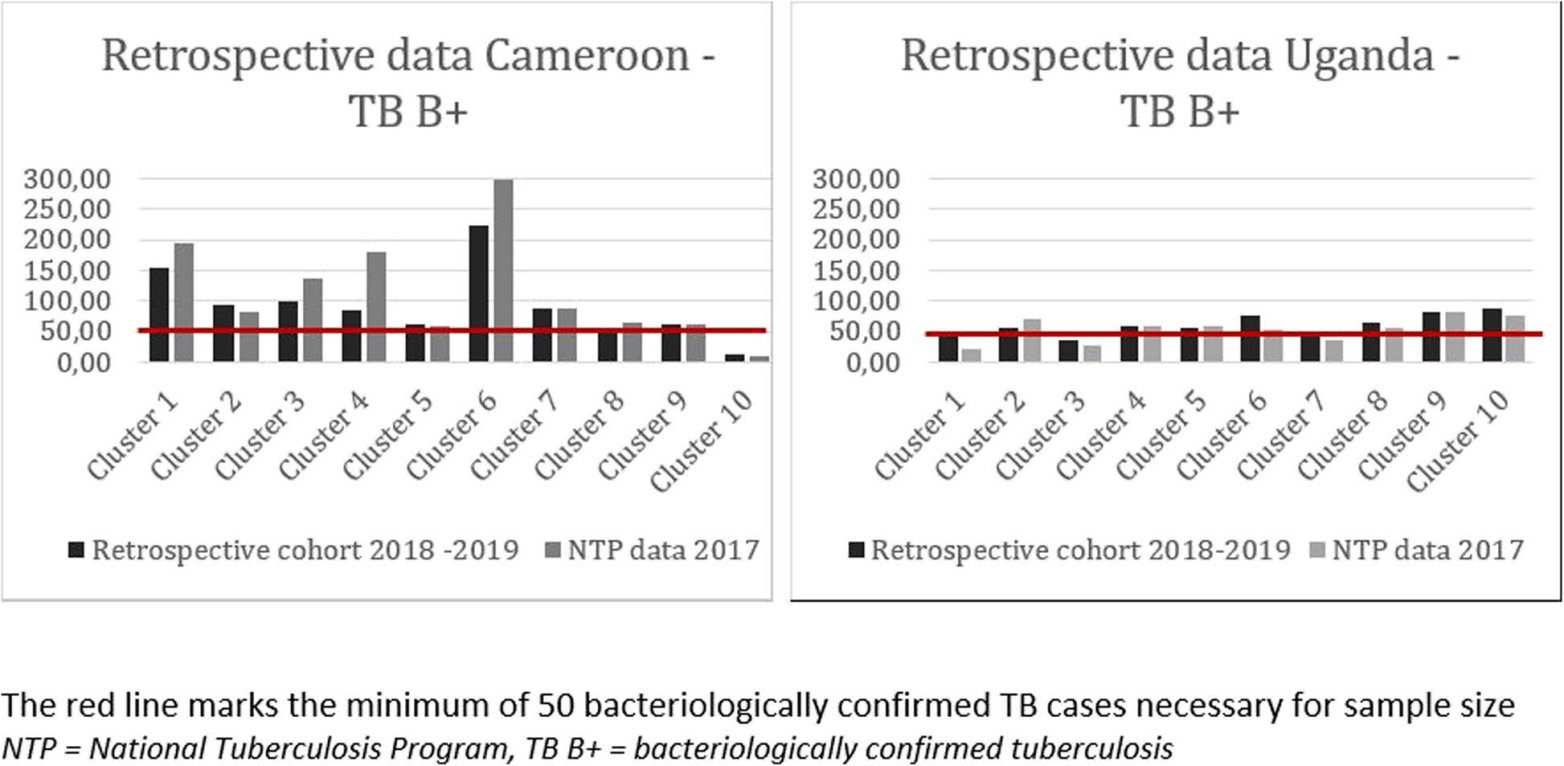
Retrospective data of tuberculosis bacteriologicaly confirmed cases in Cameroon and Uganda

From Fig. 1, we observed that the clusters facilities in Cameroon had enough TB patients to meet the minimum number of 50 bacteriologically confirmed TB patients per cluster per year to reach the study sample size within 12 months, except for one cluster (cluster 10). In Uganda, there were 3 clusters that did not meet the minimum of 50 bacteriologically positive TB patients (cluster 1, cluster 3, and cluster 7). Despite some fluctuations, data were consistent between the 2017 National TB Program reports and data collected retrospectively from TB registers between April 2018 and March 2019.

### Acceptability

The team conducted 11 FGD with 42 men and 32 women. The mean FGD duration was 105 min for women and 128.5 min for men. One FGD with women in the Littoral region of Cameroon was not done as the required minimum number of 6 participants was not reached. Twenty-four IDI were conducted with providers and community leaders (Additional file 3). We discussed contextual and perceived barriers to facility-based TB child contact management, perceived benefits of a community intervention, and prerequisites for its implementation.

### Barriers to facility TB screening

The main reasons cited by TB patients for not bringing their children to the health facility were financial, sociocultural, or stigma-related. The health personnel and community leaders cited financial difficulties and shame as main reasons for patients not bringing their children to the health facility for TB screening. In addition, the TB focal person highlighted the importance of the initial encounter (or counseling) with the index case in helping patients understand TB prevention.

I have to spend 3000 for each of the eight people [his contacts] to come here at the health facility and also spend 3000 shillings to transport them back, so transport would strain me – Male FGD participant, Uganda

They [TB patients] are not coming back with the children not because they don’t want to, but because maybe they did not understand an important part [of the health education] – CHW, IDI participant, Cameroon

### Conditions for acceptability of the community intervention

The community intervention for TB screening and TPT management is considered acceptable by both TB patients, healthcare providers, and community members. Besides removing distance and related transport costs, patients noted further benefits of the household visit, including the confirmation of the child’s good health (not TB infected) and ensuring through TPT that a parent’s TB infection will not be passed to the children.

I would accept because I had it [TB] … and I need to make sure my children are healthy – female FGD participant, Cameroon

The people would welcome the idea because there are many children in the community who are at risk of tuberculosis but they have not yet received preventive therapy – male FGD participant, Uganda

From the providers’ points of view, the intervention was coherent and welcome though they questioned its sustainability. One CHW even highlighted the fact that many research projects test interventions in the communities and when they finish the project and remove the means, there is no benefit left for the community:

You [implementing organizations] come, you tell us what has to be done, you teach us what to do, it [the project] starts well and after a certain time it stops. And we don’t understand why it stopped. – CHW, IDI participant, Cameroon.

### Prerequisites for feasibility of the community intervention

Both patients, community leaders, and health staff agreed that the cornerstone of this community intervention is the explanation and the counseling offered by the TB focal person at the first visit with the index case. During this visit, TB education should be done, rapport should be created through demonstrating empathy, providing options, and ensuring confidentiality.

During the first visit is when the rapport is created. Once the patient gets to know that you are friendly and you will keep their information, you will not release it to any other person; through my experience, these clients are willing to welcome you to their homes – TB focal person, IDI participant, Uganda

Generally, FGD participants preferred trained CHWs who are polite and explain well all activities that will take place. There was no preference for gender, as long as the person is well trained. An essential point discussed only by the health staff and community leaders is the CHWs’ motivation, which is an element which is always present in the discussions with community leaders and healthcare providers.

If we have enough staff and there are [financial] resources, it [TB screening] can be improved – community leader, IDI participant, Uganda

Detailed results of the intervention acceptability are presented in the Additional file 4.

### Adaptation and integration

#### TB services and available tools

In all cluster facilities, contact investigation was done by a health care worker (nurse, clinician), for children < 5 years in both countries. HIV-positive contact children were also screened in Uganda. Both countries had registers recording child contacts initiated on TPT and their TPT outcome. At the time of assessment, National TB Programs were about to introduce in both countries a contact screening register to record all household contacts per index cases with the results of their TB screening. None of the two countries had tools to monitor TPT adherence and tolerability. In Uganda, TB contact screening could be done at community level by the facility TB focal person. In practice, this activity was done only with support from implementing partners to cover transport cost. At the time of the site assessment, no registered data were available about the number nor age of household child contacts in both countries.

Six months isoniazid prophylaxis was used in all facilities and was delivered monthly at the facility by the TB focal person. All study facilities were expecting to introduce the 3 months isoniazid rifampicin (3RH) TPT under the CaP TB Project. TB screening, clinical and microbiological diagnosis for children with presumptive TB and drugs and treatment monitoring were free of charge. Families had to pay for further TB investigations like chest radiography (CXR). Drug-resistant cases and complicated cases were referred to higher-level health facilities. HIV testing was provided at the health facility in all study sites, in close collaboration but in separate units of the same department in Cameroon with the exception of two clusters facilities where TB and HIV services were fully integrated, and integrated in the same department in Uganda. In all study sites TB drugs were stored at the TB clinic at room temperature in a locked cabinet and in two health facilities at the facility pharmacy. A reference and counter-reference system between the CHW and the PHC staff or higher-level health facilities was set in the Ugandan clusters but almost inexistant or not functional in the Cameroon clusters.

Table 2 below summarizes practices and available tools under the standard of care in the two countries.

**Table 2:** Practices and tools in the routine system

Activity	Cameroon	Uganda
Index case identification	By the TB focal person at the health facility using the TB register	By the TB focal person at the health facility using the TB register
Contact investigation	At the health facility. Contact tracing register about to be introduced	Possibility of household contact investigation by the TB focal person Contact register about to be introduced
Symptom screening	At the health facility, no tool	Possibility of household screening, intensified case finding tool (checklist)
HIV testing of child contacts	Only medical personnel at the health facility	Possibility of HIV testing by CHWs or healthcare staff
TPT initiation	6H, at the health facility, recorded in the TPT register by the TB focal person	6H, at the health facility, recorded in the TPT register by the TB focal person
TPT follow-up: adherence and tolerability	Adherence and tolerability not assessed. No tool for TPT adherence. TPT register used for follow-up at the health facility	Adherence and tolerability not assessed. No tool for TPT adherence. TPT register used for follow-up at the health facility
Safety management	At facility. No tool for safety evaluation	At facility. No tool for safety evaluation
TPT outcome assessment	According to national TB guideline: completed, death, lost to follow-up. At the health facility by the TB focal person	According to national TB guideline: completed, death, lost to follow-up. At the health facility by the TB focal person
TB diagnosis	TB investigations at the health facility or referral at a higher-level facility Available tools: chest X-ray, sputum collection, nasopharyngeal aspirate, XpertMTB/RIF testing Laboratory results in the lab register	TB investigations at the health facility or referral at a higher-level facility Available tools: chest X-ray, sputum collection, XpertMTB/RIF testing. Laboratory data collected in the lab register

*H* isoniazid, *TB* tuberculosis, *TPT* tuberculosis preventive therapy

#### Checking data quality

A total of 1091 TB patients, out of which 708 were bacteriologically confirmed, have been registered between May 1^st^, 2018, and Oct 31^st^, 2018, in the TB registers of the cluster sites. The overall median rate of missing data was 0.3% (interquartile range (IQR) [0–3%]) in Cameroon, ranging from 0 to 8.6% and 0.4% (IQR [0–0.6%]) in Uganda, ranging from 0 to 1.4%. The median error rate was 1.1% (IQR [0.6–1.4%]) in Cameroon, ranging from 0.3 to 3.6% and 0.0% (IQR [0–0%]) in Uganda. The biggest rate of missing data was for the registration date, with a maximum of 8.2% in cluster 6. The biggest rate of errors was for the type of TB, with a maximum of 2.1% in cluster 5 (see Additional file 5).

#### Resources and procedures for the community intervention

The type of human resources at facility level was similar in the two countries. The community intervention involved mainly TB focal persons and in addition, one clinician was identified as a safety monitor for referred children with TPT tolerability concerns and was trained for safety assessment. Regarding CHWs, in Uganda, village health teams were already involved in TB activities at facility level within the CaP TB project (called linkage facilitators). It was proposed to identify CHW for the community intervention among the linkage facilitators. In Cameroon, since there was no CHW involved in TB activities, they were identified among existing CHW involved in other community health activities (COSA—Comité de Santé (health committee)). Based on a literature review of community interventions [14, 17, 34–37], findings of the acceptability survey and discussion with stakeholders in both countries, a procedure for selection of the CHW was proposed including the following criteria: having experience with community work, living in the same community, medium level of education, time to perform the tasks, accepted and respected by the communities. In Cameroon, there were 3 CHW per intervention site, with a total of 15 CHW and in Uganda, there were 2 CHW per intervention site for HC IV and one CHW for HC III, with a total of 12 CHW. In both countries, it was proposed that CHW will report to the facility TB focal person.

Taking into consideration the absence of research experience of CHW, to guarantee good quality of data and to ensure that a clear distinction could be made between activities related to the intervention and activities related to research, it was proposed that RAs will accompany CHWs to households and will be in charge of the informed consent procedure for contacts and data entry in the electronic case report form (eCRF) from source documents filled by the CHW.

Transport cost for the community activities was identified as a barrier by both TB patients and providers in the acceptability survey and by stakeholders during study preparation. It was proposed that the study will cover the transport cost but that existing public transport will be used as much as possible to ensure the sustainability of the intervention and avoid stigmatization. Good communication between the facility TB focal person and the CHW was also identified as a very important factor, justifying the allowance of a small budget for communication (airtime). Therefore, to ensure sustainability and to comply with existing practices, it was proposed that CHWs will not receive a salary, but will be compensated for their time and transport.

Finally, working with CHW on a new intervention implied to develop simple tools and check lists for TB symptom screening, adherence, and tolerability assessment. These tools were developed in coordination with country TB stakeholders (see Additional file 6 for symptom screening checklist). These tools were incorporated in simple standard operating procedures used for the training of the CHWs. CHWs were also trained to recognize potential severe symptoms or signs related to other diseases than TB that would justify urgent referral of the child to the facility. Although the initial aim of the study was that CHW would initiate child contact son TPT in the household, both National TB Programs of Cameroon and Uganda requested that initiation would be done by a nurse in the household and that the CHW would be in charge of the follow-up on his own. They also requested a more frequent follow-up by the CHW, 1 and 2 weeks after initiation instead of 4 weeks as done at facility by the TB focal person.

In Cameroon, due to national guidelines, HIV testing could only be performed by a nurse; therefore, HIV testing in the community was done by a nurse. Cascade training was organized by the country research team in each cluster facility sites followed by supervision by the RAs.

## Discussion

### Main results and implications for implementation

The results of this feasibility study brought important information for the implementation of the CONTACT study which was initiated in October 2019.

The qualitative results showed that community activities were well accepted by beneficiaries and healthcare providers alike in both countries. The emerging barriers to health facility TB contact investigation and TPT management were coherent with the literature findings and support the proposed community intervention [10, 38, 39]. A study conducted in Uganda identified the following barriers to TB contact investigation: stigma, limited knowledge about TB among contacts, insufficient time and space in clinics for counseling, mistrust of health center staff among index patients and contacts, and high travel costs [39]. Our qualitative assessment identified similar barriers for child TB contact investigation. Stigma and disclosure play an essential role firstly in the diagnosis of TB patients and secondly in the acceptability of the community intervention. Stigma and disclosure influence how TB patients accept a team coming to their household for TB contact investigation. Both beneficiaries and providers insisted on the importance of proper selection, training, and support of CHW in charge of the household visits and how important it is to build confidence with beneficiaries. Although CHW have been involved in TB community activities, their involvement in the TPT management was quite unique in the CONTACT study [34, 15].

**Fig. 2:**
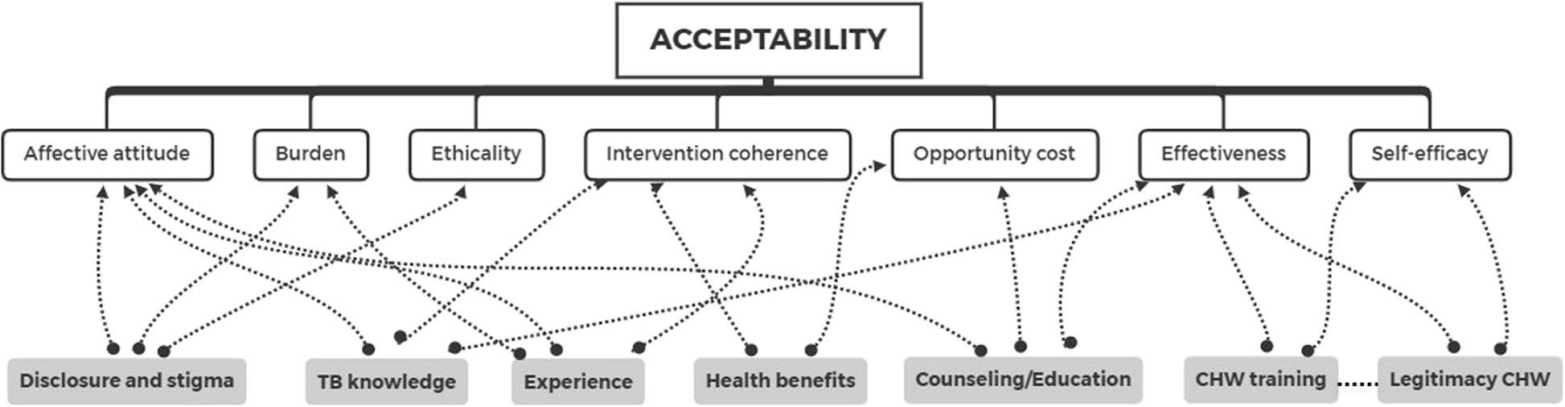
Acceptability components and emerging themes

A deductive approach for acceptability based on the acceptability framework proposed by Sekhon et al. [40] combined with inductive theorizing can be used to propose a model which is specific for community TB investigation and TPT management, as illustrated in Fig. 2. This framework contains 7 concepts: burden, affective attitude, ethicality, intervention coherence, opportunity cost, effectiveness, and self-efficacy [34]. Concepts like TB stigma and disclosure that emerged from the discussions relate to the affective attitude, burden, and ethicality components of acceptability [41, 42]. TB knowledge and experience with other community activities influence the affective attitude of the participants toward the intervention and reveals the coherence of the community intervention [43]. Nevertheless, through experience from other community activities, participants anticipate the burden this kind of intervention could represent [37]. TB patients anticipated the added health benefit of a community intervention through the possibility of discussing other health problems, which are related to the intervention coherence and the opportunity cost. Initial counseling and TB education for the index case is essential for acceptability and in the proposed framework these aspects influence the affective attitude of the participants, the opportunity cost, and the effectiveness of the intervention. Finally, CHW legitimacy and training have a role to play in the effectiveness of the intervention and in the self-efficacy component of acceptability [44].

The findings of the qualitative assessment were used to formulate recommendations on recruitment of CHWs, training curricula for CHWs, adapt team transportation for field visits, and concentrate efforts on key elements that were important to the participants (like preparing the initial visit). Financial and non-financial means are known to improve performance of CHWs for the community activities [36], and close attention is paid to CHW competence and training. Kok et al. identified the specific activities that led to a better performance of CHWs and frequent supervision and continuous training were main influencers [35]. The CONTACT study ensured both these elements by close supervision of the CHWs by the research assistants and TB focal persons and job mentorship by TB focal persons. It was indeed very important to ensure good communication and linkages between CHW and TB focal persons and empowering CHWs in performing their activities, as dully noted in the Astana declaration on primary health care: “Investment must encompass the empowerment of individuals and communities, with recognition of the importance of skills, local context and health needs” [45]. Although incentives were not provided per se, transport and communication costs were covered by the study. This is an important aspect to be taken into account for sustainability purposes, as CHW transportation needs to be ensured in order for this type of community project to succeed [45]. This funding aspect is crucial to ensure sustainability of the community intervention and will be further assessed in the cost effectiveness and process evaluation parts of the CONTACT study.

### Community intervention evaluated in the CONTACT study

Under the CONTACT study, all study drugs are kept at the health facilities under the responsibility of the TB focal person who prepares the necessary drug packages before each study visit. CHWs ensure the delivery of the drugs prepared and packed by the TB focal person to the contact children, according to the study visit procedures. The research team assessed TB services in the standard of care and the existing tools to ensure a smooth integration of study activities and source documents into current practice and to avoid disruption of routine activities by the CONTACT study. This part of the feasibility study is crucial to the sustainability of the proposed intervention beyond the end of the CONTACT study. It is challenging to integrate both the intervention and tools to evaluate the outcome of the intervention in a health system that may be weakened by lack of resources, turnover of personnel, and high workload. The burden that the proposed intervention is putting on the health providers is an essential element of knowledge to practice translation. This feasibility assessment allowed us to identify which existing tools could serve as source documents and which additional tools will need to be introduced, keeping an adaptation to each country’s specificities. Integrating research and practice is a core element of translating the proposed intervention, if proven effective, into current practice. Selecting sites from the CaP TB Project is an asset for the study because of capacity reinforcement for pediatric TB case management and improvement of data collection tools. In order to avoid any disruption of the CHWs’ activities for the delivery of the intervention by additional research tasks (consent, data entry), we allocated the research tasks to research assistants. This was also emphasized by the WHO ethics research committee at the time of first protocol submission.

The estimate of one child under 5 years per household in each country was consistent with findings by Yuen et al. who reported 0.83 (95% confidence interval (CI) 0.80–0.86) children under 5 years per household in Cameroon and 0.93 (0.89–0.96) in Uganda [27]. Therefore, the assessment of the cluster facilities’ capacity to enroll child contacts under 5 years was made using data on the number of index cases from TB registers, assuming there was one index case per household and one child contact under 5 years per index case. In the context of few available cluster sites within the CaP TB Project, the retrospective data collection of bacteriologically confirmed TB cases and comparison with National TB Program reports was extremely useful in informing the study team on potential problematic sites and adjusting the recruitment period from 12 to 15 months. This step was essential for activity planning and budget review [46].

### Limitations

TB services have been assessed through a survey and discussions with health providers; nevertheless, there was no observation of practices by the research team. Indeed, collecting data through a survey could induce a declaration bias of the person filling in the survey. However, conducting observations would have been limiting for the feasibility study as some health facilities have very low patient flows, meaning the events to be observed would be rare. In addition, it is well known that observations could induce the Hawthorne effect, when subjects perform better because they know they are observed [47].

It is worth mentioning that during the qualitative assessment, participants could have been inclined to declare that they did certain activities because of social desirability bias, meaning they wanted to be perceived in a positive way by the researchers [48]. The team tried to minimize this bias by always reassuring the participants that there is no right or wrong answer, that the discussions were not part of an evaluation by their hierarchy, and by using the technique of indirect questioning (i.e., “Why don’t people in general bring children back to the health facility for TB screening?”).

## Conclusion

This study has identified a feasible and acceptable community intervention for TB screening and TPT management for further evaluation in the context of two high TB burden and resource-limited countries. All activities occurring after the start of inclusions are assessed under an ongoing process evaluation that will support the interpretation of the CONTACT study effectiveness. Capturing what is delivered in practice can enable the researchers to distinguish between the adaptations made to fit different contexts and changes that undermine intervention fidelity altogether [49]. In addition, a qualitative assessment of post-intervention acceptability by child contact parents, facility personnel, CHWs, and stakeholders will be done at the end of the study and a cost-effectiveness evaluation will be performed from both a provider and societal perspectives.

## Supplementary Information

**Additional file 1.** CONTACT pre-intervention timeline.

**Additional file 2.** Cluster assessment form.

**Additional file 3.** Qualitative participants characteristics.

**Additional file 4.** Detailed qualitative results.

**Additional file 5.** Missing rates and Error rates.

**Additional file 6.** Symptom Screeening checklist.

## Data Availability

The datasets used and/or analyzed during the current study are available from the corresponding author on reasonable request. The survey used for TB services is found in the Additional file 1.

## Abbreviations

CaP TB: Catalyzing Pediatric Tuberculosis Innovations
CHW: Community health worker
CXR: Chest radiography
DHS: Demographic health survey
FGD: Focus group discussion
H: Isoniazid
IDI: In-depth interview
TB: Tuberculosis
TPT: Tuberculosis preventive therapy
WHO: World Health Organization
