# Supplementary figures and images for: Feasibility of a randomized clinical trial evaluating a community intervention for household tuberculosis child contact management in Cameroon and Uganda
Source: Pilot Feasibility Stud. 2022 Feb 11;8:39. doi: 10.1186/s40814-022-00996-3 (PMC8832743; doi:10.1186/s40814-022-00996-3)

**Additional File 1**

CONTACT study pre-intervention timeline


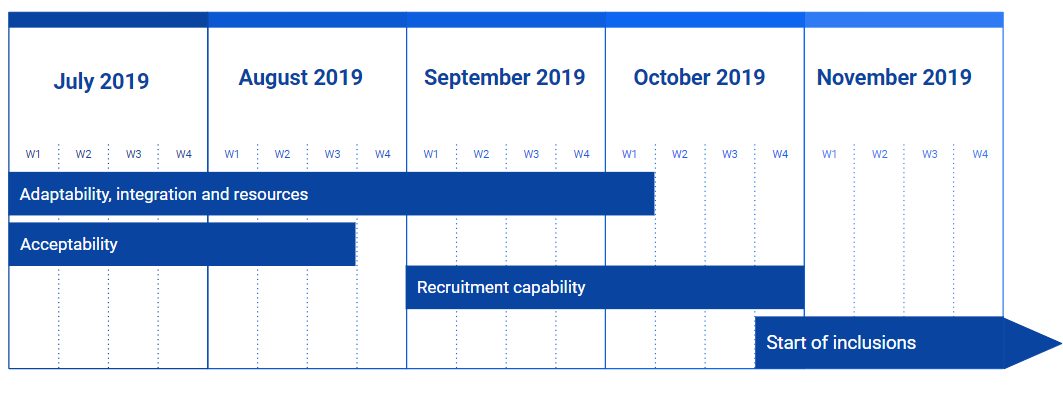

Supplement: Supplementary file 1 — Additional file 1. CONTACT pre-intervention timeline. [file 40814_2022_996_MOESM1_ESM.docx]
