# Supplementary material for: Feasibility of a randomized clinical trial evaluating a community intervention for household tuberculosis child contact management in Cameroon and Uganda
Source: Pilot Feasibility Stud. 2022 Feb 11;8:39. doi: 10.1186/s40814-022-00996-3 (PMC8832743; doi:10.1186/s40814-022-00996-3)
Supplement: Supplementary file 2 — Additional file 2. Cluster assessment form. [file 40814_2022_996_MOESM2_ESM.docx]

**Additional File 2**

*Cluster assessment form*

CONTACT TRACING AND MANAGEMENT

District name: __________________________________

Facility name: _________________________________

1. Who are the child contacts screened? Child contacts living in the same household, index case children only, child contacts < 5 years, HIV+ child contacts, none

__________________________________________________________________________________

a. Where is recorded the information about identified contacts? Form, log, note book, no records

_____________________________________________________________________________________

b. Who is in charge of following up with TB index case and make sure children living in his/her household are brought to the facility for screening?

_____________________________________________________________________________________

c. How is the follow-up done? (Phone calls, SMS…)

___________________________________________________________________________________

2. Who is in charge of the screening of contacts?____________________________________________

a. Is screening performed using a standardized questionnaire?

_____________________________________________________________________________________

b. Is result of TB screening recorded? If yes where?

_____________________________________________________________________________________

3. Who does the prescription of preventive therapy and where (facility/community)? ________________________________________

a. Is there counselling about tolerability and adherence during initiation and follow-up of preventive therapy?

_____________________________________________________________________________________

b. Are there tools using to assess tolerability and adherence? (Questionnaire, visual scale, calendar…..)

_____________________________________________________________________________________

c. Who delivers the preventive therapy? TB focal person, TB nurse, pharmacy

_____________________________________________________________________________________

d. How is the treatment delivered and where (facility/community)? Individual tablets drugs blisters, boxes of drugs

_____________________________________________________________________________________

e. Is pyridoxine systematically prescribed and delivered?______________________________

4. Are there services costs/fees that the patients/caregivers must pay during the process of TB screening, clinical assessment of eligibility for IPT initiation, IPT and follow-up? If Yes, please detail

_____________________________________________________________________________________

REFERRAL SYSTEM

Number of health centres that refer children to this cluster facility: ____________________

Maximum distance (Km or time) between the furthest health centre and the cluster facility?_________

Referral from the cluster facility to reference centres

1. List the reasons for referral :____________________________________________________

_____________________________________________________________________________________

2. Is there a referral form? Type of document (form, log, register…) and MOH or facility form

_____________________________________________________________________________________

If yes, is a copy kept at the cluster facility?___________________________________________

3. Is there a direct communication between the cluster facility and the referral centre? (telephone, SMS, other):

____________________________________________________________________________________

4. Does the cluster facility receive feedback from the referral centre? (how?)

____________________________________________________________________________________

Referrals from peripheral health centres to the cluster facility

5. Is there a referral form? Type of document (form, log, register…) and MOH or facility form

____________________________________________________________________________________

If yes, does the cluster facility keep a copy of the referral? Which document (form, log,…)

____________________________________________________________________________________

6. Does the cluster facility send a feedback to the centre?___________________________________

7. Is the cluster facility contacted by the referral centre before referral? How (telephone, SMS):

__________________________________________________________________________________

8. Does the cluster facility involve the peripheral centres for contact screening? If yes, how?

____________________________________________________________________________________

9. Are there meetings between the cluster facility and peripheral centres? frequency?

____________________________________________________________________________________

TB/HIV INTEGRATED SERVICES

Prevention and treatment of HIV-infected child contacts

Name of district: __________________________________

Name of facility: _________________________________

1. Is there a HIV clinic in the facility ?___________________________________ ; if yes

a. What is the relation between TB and HIV clinics? (same department, same clinician, none)

______________________________________________________________________________

b. Which clinic is in charge of HIV for TB-HIV co-infected patients?

____________________________________________________________________________________

2. Are children tested for HIV at the TB clinic? ___________

If yes, who is tested? (Different options possible)

❑ All patients with presumptive TB

❑ All patients with TB diagnosis

❑ All child contacts

❑ Only child contacts of HIV+ TB index cases

❑ Other (detail) :________________________________________________

3. Where is done the TB screening of HIV+ child TB contacts? ❑ TB clinic ❑ HIV clinic

4. Where is initiated preventive therapy in HIV+ child TB contacts? ❑ TB clinic ❑ HIV clinic 5. Where is done the preventive therapy follow-up of HIV+ child TB contact? ❑ TB clinic ❑ HIV clinic

6. Where is filled the preventive therapy register for HIV+ child TB contacts? ❑ TB clinic ❑ HIV clinic

7. Where is done the TB diagnosis of HIV+ child TB contacts? ❑ TB clinic ❑ HIV clinic

8. Where are registered HIV+ child TB contacts with TB? ❑ TB clinic ❑ HIV clinic

9. How and when is initiated preventive therapy and ART for HIV+ child TB contacts __________________________________________________________________________________

10. At the HIV clinic if an HIV+ adult is diagnosed with TB:

a. Where is TB treatment initiated? ❑ TB clinic ❑ HIV clinic

b. Where is registered the patient? ❑ TB clinic ❑ HIV clinic

c. Where is done the screening of contacts? ❑ TB clinic ❑ HIV clinic

MANAGEMENT OF ANTI-TUBERCULOSIS DRUGS

1. If the preventive therapy is delivered at the TB clinic

a. When and how is organised the supply of preventive therapy? Frequency (fixed days or when needed) and procedure of request

______________________________________________________________________________

b. Is there a stock management? describe the stock cards or logs

_____________________________________________________________________________

______________________________________________________________________________

c. Where and how are stored the drugs? In terms of security, temperature, humidity

______________________________________________________________________________

2. At the facility pharmacy

a. Is there a stock management? ask to see the stock cards or logs

_____________________________________________________________________________

b. Where and how are stored the anti-tuberculosis drugs? Security conditions (locked key board, cool area, humidity )

_____________________________________________________________________________

Name and function of the person collecting the information: ____________
