# Supplementary material for: Feasibility of a randomized clinical trial evaluating a community intervention for household tuberculosis child contact management in Cameroon and Uganda
Source: Pilot Feasibility Stud. 2022 Feb 11;8:39. doi: 10.1186/s40814-022-00996-3 (PMC8832743; doi:10.1186/s40814-022-00996-3)
Supplement: Supplementary file 3 — Additional file 3. Qualitative participants characteristics. [file 40814_2022_996_MOESM3_ESM.docx]

**Additional File 3**

*Characteristics of participants and discussion duration for FGD and IDI*

| Country |  | FGD participants | | | |  | IDI participants |
| --- | --- | --- | --- | --- | --- | --- | --- |
|  | Male | | Duration  (min) | Female | Duration  (min) | |  |
| **Cameroon** |  | |  |  |  | |  |
| Cluster 1 SOC model (Centre) | 7 | | 92 | 5 | 71 | | 4 |
| Cluster 2 ITV model (Centre) | 8 | | 100 | 6 | 141 | | 4 |
| Cluster 3 SOC model (Littoral) | 7 | | 127 | -* | - | | 4 |
| Cluster 4 ITV model (Littoral) | 7 | | 101 | 7 | 134 | | 4 |
| **Uganda** |  | |  |  |  | |  |
| Cluster 1 SOC model | 6 | | 144 | 8 | 104 | | 4 |
| Cluster 2 ITV model | 7 | | 113 | 6 | 106 | | 4 |
| **TOTAL** | **42** | | **677** | **32** | **556** | | **24** |

*one FGD in the littoral region could not be done due to lack of participants
