# Supplementary material for: Feasibility of a randomized clinical trial evaluating a community intervention for household tuberculosis child contact management in Cameroon and Uganda
Source: Pilot Feasibility Stud. 2022 Feb 11;8:39. doi: 10.1186/s40814-022-00996-3 (PMC8832743; doi:10.1186/s40814-022-00996-3)
Supplement: Supplementary file 4 — Additional file 4. Detailed qualitative results. [file 40814_2022_996_MOESM4_ESM.docx]

**Additional File 4**

**Detailed qualitative results**

*Barriers to facility-based TB screening*

All TFP in both countries stated they ask TB patients to bring back their children to the health facility for TB screening as requested by national TB guidelines. In their overall assessment based on experience, patients’ adherence to such a request is poor. During the FGDs, some TB patients confirmed they were indeed requested to bring their children to the facility for TB screening, among them, few did comply. Others declared that they were never asked to bring their children to the health facility for screening, stating they might have done so if requested.

The main reasons cited or anticipated by TB patients for not bringing their children to the health facility were financial, sociocultural or stigma-related. As illustrated below, facility-based child contact TB screening carries financial costs that make child-contact screening less financially and geographically accessible, especially transport costs for those living far away from the facility and those who have many children <5 years to bring for screening and more generally, the poor. This is corroborated by health personnel who did understand parents may face challenges when they cannot afford the transport cost and other costs such as buying food for children while waiting for the screening procedures.

*“I need to get transport to transport about 8 people to come back and forth. For starters coming here and going back home alone, they take me for 3000 shillings. So I have to spend 3000 for each of the eight people to come here at the health facility and also spend 3000 shillings to transport them back, so transport would strain me”* – Male participant, Uganda

*“When you ask a parent to transport his 5 kids and bring them to the health center, he’s going to ask: “Are you paying for transport?””* – CHW, Cameroon

While these practical and objective facts played a role in parents’ non adherence to healthcare providers’ recommendations, some FGD participants believed non-adherence was rather a form of child neglect and pointed at the sociocultural aspects such as gender roles in childcare religion that prevent men from attending health centers when a child is sick. Traditional beliefs about diseases and TB causation (witchcraft) associated with mistrust in “modern medicine” or confidence in traditional medicine or religious prayers, negatively and strongly influence help- and health-seeking behaviors and trajectories, as well as treatment itineraries.

*“You know, African families really like going to healers in the neighborhood. He [the TB patient] will look for herbs before going to the hospital. We, African families, love the healers.” –* male participant - Cameroon

*“There are some people who think that they have been bewitched especially when that person has a persistent cough. The person may even start to blame the neighbor whom they had a quarrel with for being responsible for the cough”* – Community leader, Uganda

Whether associated to HIV or a stand-alone determinant of TB non-disclosure, TB stigma was ever-present in all FGDs and emerged as a barrier to TB health facility-based screening. Overt stigma was of particular concern and played an important role in how patients live with the disease.

*“People fear to disclose that they have TB because they might lose their jobs, they might lose their relationships; it may cause people to be isolated”* – male participant, Uganda

*“They think this [TB] is a shameful disease and that people will mock them”* – male participant, Cameroon

*“After I was diagnosed with TB, my husband threatened me “I do not want to hear anyone discussing this with anyone.” And I have never told anyone […] and it hurts me inside”* – female participant, Uganda

Healthcare providers shared similar views regarding financial shortcomings, stigma and sociocultural norms. . Indeed, provision of TB literacy and the rapport that is built during this initial visit are essential for a good follow-up during treatment. When such initial visit is rushed due to a high workload and patients queuing at the health center, the information is not passed to the patient and in consequence, this important step is overlooked. As explained in one instance by a doctor and facility manager, health worker’s priority is to cure patients who present themselves at the facility, not to manage the contacts.

“*They [TB patients] are not coming back with the children not because they don’t want to, but because maybe they did not understand an important part [of the health education]”* – CHW, Cameroon

*“The health facility is overwhelmed by the services here. You get here and you find many people here and that means that on that day you will not be attended to and might need to return the following day. That means that you will have lost two days of work, the child has also missed school. And you know many of us earn an income by the day”* – male participant, Uganda

*Conditions for acceptability of a community intervention*

From the patients’ perspectives the proposed intervention (19) was acceptable and made sense in both countries, as it will be helpful to many in overcoming the main barriers to facility-based child-contact TB screening, and in particular, transport costs that many TB patients cannot afford.

Besides removing distance and related transport costs, patients noted further benefits of the household visit, including the confirmation of the child’s good health (not TB infected) and ensuring through TPT that a parent’s TB infection will not be passed to the children.

*“I would accept because I had it [TB]… and I need to make sure my children are healthy” –* female patient, Cameroon

Additionally, parents get the opportunity to address other health or environmental problems with the CHW that might come up during the home visit discussions.

*“Many people cannot afford to go to the health facility. When you go to the home, you can teach many things and they are able to know and understand better rather than spend the whole day at the health facility with a child in the back”* – CHW, Uganda

All participants welcomed the community intervention. Only one male TB patient participant in Cameroon stated he preferred taking his children to the health center for TB screening because all investigations are available there as opposed to the limited knowledge or diagnostic means in the community intervention model. Indeed, the discourses of some other participants showed the obstacles they went through from district hospitals to the capital city on their diagnosis itinerary.

Elsewhere, many participants raised concerns about unintended disclosure and subsequent stigma from the other community members following home visits. The proposed approach to home visit did not include enquiries and screening of other children <5 years living in the same compound, participants were ambivalent when asked about the opportunity to also screen such children as the risk of stigma increases. Those who would agree to the screening of children playing together with their own children, preferred to inform their parents themselves based on past positive relationship and their living together experience.

*“They [the family of the index case] need to check if the neighbors are not sick. It will be difficult, but if the family has accepted their fate [having TB], they can help the others [the neighbors] accept as well”* – CHW, Cameroon

“*Some clients do not want their neighbors to know because they know that the moment these know that they are having TB, they will either be chased from their place where they are renting or sometimes they may be isolated*” – TB focal person, Uganda

From the providers’ points of view, the intervention was coherent and welcome though they questioned its sustainability.

One CHW even highlighted the fact that many research projects test interventions in the communities and when they finish the project and remove the means, there is no benefit left for the community:

*“You [implementing organizations] come, you tell us what has to be done, you teach us what to do, it [the project] starts well and after a certain time it stops. And we don’t understand why it stopped.”* – CHW, Cameroon.

*Prerequisites of feasibility of community TB screening and TPT management*

Both patients, community leaders and health staff agreed that the cornerstone of this community intervention is the explanation given and the counseling offered by the TFP at the first visit with the index case. During this visit, TB education should be done, rapport should be created through demonstrating empathy, providing options, and ensuring confidentiality.

“*During the first visit is when the rapport is created. Once the patient gets to know that you are friendly and you will keep their information, you will not release it to any other person; through my experience, these clients are willing to welcome you to their homes”* – TB focal person, Uganda

This was echoed by healthcare personnel, especially in Uganda, where TFP and CHWs stressed successful implementation depends on thorough information given to the index case. Of paramount importance is the quality of the initial counseling, and therefore on CHWs’ training, experience and acquired legitimacy.

*“I think that if we explain very well to the patient, it will be acceptable. […] when it is well explained, the patient understands the benefit [of the community intervention] from the explanation” –* health facility manager, Cameroon

*“At the beginning I told you, if you give them information on the initiation day, if you give them the information, they can give the medicine [to their children].”* – TB focal person, Uganda

The opinions of the patients converged towards their need to be informed well in advance of the timing of the home visit so that they can ensure that their children are at home at the time of the visit or that they have informed their partner -if they have not yet done so- or relevant people based on need-to-know. These conditions provided, the team is welcome to come and perform the screening and even take time to discuss other family health needs or concerns.

Creating rapport was also essential for CHW and they should be trained on this subject. The legitimacy and training of a CHW was also an important determinant of feasibility according to patients and stakeholders. Some respondents and interviewees were skeptical regarding the implication of CHWs because of past negative experiences with careless CHWs who would spread information about people they visit, which shows a lack of confidentiality.

Generally, FGD participants preferred trained CHWs who are polite and explain well all activities that will take place. There was no preference for gender, as long as the person is well trained.

“*The most important thing is if you send a health worker with the expertise in whatever he or she is going to do. It does not matter if it is a doctor or a nurse, as long as they have the expertise in whatever they are going to do. It does not matter whether the health worker is male or female*” – male participant, Uganda

Even with the best intentions in mind, unintentional disclosure cannot be totally ruled out due to the specific local context, and some participants expressed their concern. Others were not at all worried about disclosure and subsequent consequences.

“*They [the neighbors] will maybe spread this [the information] everywhere. “Oh, she has this, she has that”. It’s difficult, it’s very difficult [to disclose]”* – female participant, Cameroon

*“For me it’s not a problem. It’s my house, my family”* – female participant, Cameroon

An essential point discussed only by the health staff and community leaders is the CHWs’ motivation. This is a term historically referring to money used to compensate CHWs for their time and transport. Motivation is always requested when doing any kind of research activity in both countries as highlighted by the qualitative assessment:

“*If we have enough staff and there are [financial] resources, it [TB screening] can be improved*” – community leader, Uganda

*“If there is motivation, they [the CHW] will do the work”* – community leader, Cameroon
