# Supplementary material for: Feasibility of a randomized clinical trial evaluating a community intervention for household tuberculosis child contact management in Cameroon and Uganda
Source: Pilot Feasibility Stud. 2022 Feb 11;8:39. doi: 10.1186/s40814-022-00996-3 (PMC8832743; doi:10.1186/s40814-022-00996-3)
Supplement: Supplementary file 5 — Additional file 5. Missing rates and Error rates. [file 40814_2022_996_MOESM5_ESM.docx]

## Additional File 5

*Missing rates for TB register checks*

| Country | Cluster | Registered TB patients | Bacteriologically confirmed TB | date | register number | sex | age | type of TB | type of patient | HIV status | Missing total | Missing rate total |
| --- | --- | --- | --- | --- | --- | --- | --- | --- | --- | --- | --- | --- |
| Cameroon | Cluster 1 | 113 | 75 | 0 | 0 | 0 | 0 | 1 | 0 | 0 | 1 | 0,1% |
|  | Cluster 2 | 85 | 51 | 0 | 0 | 0 | 0 | 0 | 0 | 1 | 1 | 0,2% |
|  | Cluster 3 | 75 | 52 | 2 | 0 | 0 | 0 | 0 | 0 | 0 | 2 | 0,4% |
|  | Cluster 4 | 98 | 42 | 23 | 0 | 0 | 0 | 0 | 0 | 1 | 24 | 3,5% |
|  | Cluster 5 | 40 | 32 | 0 | 0 | 0 | 0 | 0 | 0 | 0 | 0 | 0,0% |
|  | Cluster 6 | 157 | 101 | 90 | 1 | 0 | 0 | 0 | 1 | 2 | 94 | 8,6% |
|  | Cluster 7 | 51 | 36 | 0 | 0 | 0 | 0 | 0 | 0 | 0 | 0 | 0,0% |
|  | Cluster 8 | 40 | 27 | 0 | 0 | 0 | 0 | 0 | 0 | 4 | 4 | 1,4% |
|  | Cluster 9 | 46 | 29 | 3 | 2 | 0 | 0 | 0 | 0 | 7 | 12 | 3,7% |
|  | Cluster 10 | 19 | 9 | 0 | 0 | 0 | 0 | 0 | 0 | 0 | 0 | 0,0% |
| Uganda | Cluster 1 | 30 | 14 | 0 | 0 | 0 | 0 | 0 | 0 | 0 | 0 | 0,0% |
|  | Cluster 2 | 32 | 25 | 0 | 0 | 0 | 0 | 0 | 0 | 0 | 0 | 0,0% |
|  | Cluster 3 | 22 | 6 | 0 | 0 | 0 | 0 | 0 | 0 | 1 | 1 | 0,6% |
|  | Cluster 4 | 48 | 23 | 2 | 0 | 0 | 0 | 0 | 0 | 0 | 2 | 0,6% |
|  | Cluster 5 | 37 | 28 | 0 | 0 | 0 | 0 | 0 | 0 | 0 | 0 | 0,0% |
|  | Cluster 6 | 28 | 28 | 0 | 0 | 0 | 0 | 0 | 0 | 1 | 1 | 0,5% |
|  | Cluster 7 | 7 | 7 | 0 | 0 | 0 | 0 | 0 | 0 | 0 | 0 | 0,0% |
|  | Cluster 8 | 35 | 32 | 0 | 0 | 0 | 0 | 1 | 0 | 0 | 1 | 0,4% |
|  | Cluster 9 | 62 | 49 | 6 | 0 | 0 | 0 | 0 | 0 | 0 | 6 | 1,4% |
|  | Cluster 10 | 66 | 42 | 2 | 0 | 0 | 0 | 0 | 0 | 0 | 2 | 0,4% |

*Error rates for TB register checks*

| Country | Cluster | Registered TB patients | Bacteriologically confirmed TB | date | register number | sex | age | type of TB | type of patient | HIV status | Error rate total | Error rate total |
| --- | --- | --- | --- | --- | --- | --- | --- | --- | --- | --- | --- | --- |
| Cameroon | Cluster 1 | 113 | 75 | 0 | 0 | 0 | 0 | 8 | 3 | 0 | 11 | 1,4% |
|  | Cluster 2 | 85 | 51 | 0 | 0 | 0 | 0 | 1 | 6 | 0 | 7 | 1,2% |
|  | Cluster 3 | 75 | 52 | 0 | 0 | 0 | 0 | 1 | 4 | 0 | 5 | 1,0% |
|  | Cluster 4 | 98 | 42 | 2 | 0 | 0 | 0 | 0 | 0 | 0 | 2 | 0,3% |
|  | Cluster 5 | 40 | 32 | 2 | 0 | 0 | 0 | 6 | 2 | 0 | 10 | 3,6% |
|  | Cluster 6 | 157 | 101 | 0 | 0 | 0 | 0 | 6 | 0 | 0 | 6 | 0,5% |
|  | Cluster 7 | 51 | 36 | 0 | 0 | 0 | 0 | 0 | 4 | 0 | 4 | 1,1% |
|  | Cluster 8 | 40 | 27 | 0 | 0 | 0 | 0 | 1 | 0 | 0 | 1 | 0,4% |
|  | Cluster 9 | 46 | 29 | 0 | 0 | 0 | 0 | 2 | 2 | 0 | 4 | 1,2% |
|  | Cluster 10 | 19 | 9 | 2 | 0 | 0 | 0 | 0 | 0 | 0 | 2 | 1,5% |
| Uganda | Cluster 1 | 30 | 14 | 0 | 0 | 0 | 0 | 0 | 0 | 0 | 0 | 0,0% |
|  | Cluster 2 | 32 | 25 | 0 | 0 | 0 | 0 | 0 | 0 | 0 | 0 | 0,0% |
|  | Cluster 3 | 22 | 6 | 0 | 0 | 0 | 0 | 0 | 0 | 0 | 0 | 0,0% |
|  | Cluster 4 | 48 | 23 | 0 | 0 | 0 | 0 | 0 | 0 | 0 | 0 | 0,0% |
|  | Cluster 5 | 37 | 28 | 0 | 0 | 0 | 0 | 0 | 0 | 0 | 0 | 0,0% |
|  | Cluster 6 | 28 | 28 | 0 | 0 | 0 | 0 | 0 | 0 | 0 | 0 | 0,0% |
|  | Cluster 7 | 7 | 7 | 0 | 0 | 0 | 0 | 0 | 0 | 0 | 0 | 0,0% |
|  | Cluster 8 | 35 | 32 | 0 | 0 | 0 | 0 | 0 | 0 | 0 | 0 | 0,0% |
|  | Cluster 9 | 62 | 49 | 0 | 0 | 0 | 0 | 0 | 0 | 0 | 0 | 0,0% |
|  | Cluster 10 | 66 | 42 | 0 | 0 | 0 | 0 | 0 | 0 | 0 | 0 | 0,0% |
