# Supplementary material for: Feasibility of a randomized clinical trial evaluating a community intervention for household tuberculosis child contact management in Cameroon and Uganda
Source: Pilot Feasibility Stud. 2022 Feb 11;8:39. doi: 10.1186/s40814-022-00996-3 (PMC8832743; doi:10.1186/s40814-022-00996-3)
Supplement: Supplementary file 6 — Additional file 6. Symptom Screeening checklist. [file 40814_2022_996_MOESM6_ESM.docx]

**Additional File 6**

*Checklist of symptoms assessed by CHWs during TB symptom screening*

| Cough | Yes  | No  |
| --- | --- | --- |
| If yes, for how long? | > 2 weeks  | < 2 weeks  |
| Fever | Yes  | No  |
| If yes, for how long? | > 10 days  | < 10 days  |
| Lethargy/reduced playfulness/ fatigue | Yes  | No  |
| Weight loss/appetite loss or failure to thrive during the last 3 months | Yes  | No  |
| Night sweats | Yes  | No  |
| If yes, for how long? | > 2 weeks  | < 2 weeks  |
| MUAC <125mm (if <5 years) | Yes  | No  |
| Neck swelling | Yes  | No  |
| Wheeze | Yes  | No  |
| If yes, for how long? | > 2 weeks  | < 2 weeks  |
